# Citrus wastes as sustainable materials for active and intelligent food packaging: Current advances

**DOI:** 10.1111/1541-4337.70144

**Published:** 2025-03-04

**Authors:** Mirella R. V. Bertolo, Tamires S. Pereira, Francisco V. dos Santos, Murilo H. M. Facure, Fabrício dos Santos, Kelcilene B. R. Teodoro, Luiza A. Mercante, Daniel S. Correa

**Affiliations:** ^1^ Nanotechnology National Laboratory for Agriculture (LNNA) Embrapa Instrumentation Sao Carlos SP Brazil; ^2^ PPGQ, Department of Chemistry, Center for Exact Sciences and Technology Federal University of Sao Carlos (UFSCar) Sao Carlos SP Brazil; ^3^ PGrCEM, Department of Materials Engineering, Sao Carlos School of Engineering University of Sao Paulo Sao Carlos SP Brazil; ^4^ Institute of Chemistry Federal University of Bahia (UFBA) Salvador BA Brazil

**Keywords:** active packaging, citrus fruits, essential oils, intelligent packaging, phenolic compounds

## Abstract

Citrus fruits are one of the most popular crops in the world, and around one quarter of them are subjected to industrial processes, aiming at the production of different food products. Citrus processing generates large amounts of waste, including peels, pulp, and seeds. These materials are rich sources of polymers (e.g., pectin, cellulose, hemicellulose, lignin), phenolic compounds, and essential oils. At the same time, the development of food packaging materials using citrus waste is a highly sought strategy for food preservation, and meets the principles of circular economy. This review surveys current advances in the development of active and intelligent food packaging produced using one or more citrus waste components (polymers, phenolics extracts, and essential oils). It highlights the contribution and effects of each of these components on the properties of the developed packaging, as well as emphasizes the current state and challenges for developing citrus‐based packaging. Most of the reported investigations employed citrus pectin as a base polymer to produce packaging films through the casting technique. Likewise, most of them focused on developing active materials, and fewer studies have explored the preparation of citrus waste‐based intelligent materials. All studies characterized the materials developed, but only a few actually applied them to food matrices. This review is expected to encourage novel investigations that contribute to food preservation and to reduce the environmental impacts caused by discarded citrus byproducts.

## 1 INTRODUCTION

Citrus fruits (e.g., orange, lime, mandarin, and lemon) are one of the most popular crops in the world because of their pleasant taste, nutritional components, and well‐known health benefits, such as antioxidant and anti‐inflammatory properties (Panwar et al., 2021; Yun & Liu, 2022). These fruits belong to the genus *Citrus*, the largest genus of the family Rutaceae, composed of several species, each with many varieties, including spontaneous and commercial hybrids (Matheyambath et al., 2015). Plants belonging to the *Citrus* genus are often evergreen trees or shrubs with fragrant flowers, which adapt better in moderate temperatures around 25–30°C. Their fruits are classified as hesperidium, a modified berry with around eight to 16 carpels and different shapes and diameters (ranging from 4 to 15 cm), among which it is possible to find seeds and juice (Russo et al., 2021). Their peels are divided into two main parts: the colored part is the flavedo or exocarp, and the inner part is the albedo or mesocarp (generally colorless or white) (Dubey et al., 2023). The exocarp is the tissue where essential oil (EO) production and storage take place; during ripening, juice vesicles are developed from the inner epidermis, containing acids, oils, and sugars, components responsible for the characteristic flavor of citrus fruits (Russo et al., 2021). Despite these common characteristics, citrus fruits are quite diverse regarding their morphological and organoleptic attributes, such as their shape (oblate, oval, spherical, or oblong), pulp color (pale, yellow, orange, or red), size, and the shape of their seeds (Matheyambath et al., 2015).

Citrus fruits are grown all around the world, but their cultivation is concentrated in tropical and subtropical regions, such as the Americas, South Africa, Australia, Southeast Asia, and Mediterranean countries in Europe (Russo et al., 2021). In 2021, citrus fruits were the world's second most produced fruit category, with about 162 million tons. The main producing countries are China, Brazil, India, some countries of the European Union (e.g., Spain), and the United States. While China is responsible for the highest production of tangerines, Brazil is the largest producer of oranges, and India is the largest producer of lemons and limes. Oranges account for nearly half (∼47%) of the world's citrus fruits production, followed by tangerines (∼26%) and limes and lemons (∼13%) (Gonzatto & Santos, 2023). Citrus fruits are important export crops, with about 11 million tons being exported annually worldwide (among oranges, mandarins/tangerines, grapefruits, limes, and lemons), of which about 40% are oranges (Russo et al., 2021).

The vast majority of these fruits are consumed freshly, but around one quarter of all citrus fruits produced are subjected to industrial processes, aiming at the production of different food products, like juices, jams, jellies, among others (Dubey et al., 2023). This industrial processing, however, generates large amounts of waste, including citrus peels, pulp, and seeds, which together account for more than 50% of the total fruit weight and represent around 120 million tons of disposal rests per year (Teigiserova et al., 2021). Considering the difficulty of stocking citrus processing waste, it is often fed to animals, composted, or discarded in landfills or rivers, which reflects its underutilization (Yun & Liu, 2022). The high organic matter of these residues (more than 90%) and their easy fermentability lead to environmental pollution, decreasing the levels of oxygen dissolved in water (Teigiserova et al., 2021; Yun & Liu, 2022).

Despite their disposal, citrus fruit by‐products such as peels, seeds, pressed pulp, and leaves are rich sources of compounds like polyphenols, proteins, lipids, sugars, dietary fibers, vitamins, monoterpenes, organic acids, and carotenoids. The main compounds found in each of these by‐products and their quantities will depend on the species, the cultivar, the degree of ripening, and the cultivation method (Dubey et al., 2023; Russo et al., 2021).

Citrus fruit peels are mainly composed of pectin, cellulose, hemicellulose, polyphenols, and EOs. Limonene is the major compound of the EOs of citrus peels, which can be used in foods, cosmetics, and pharmaceutical products as a flavoring agent (Rafiq et al., 2018). EOs can also be obtained from citrus leaves, with different majoritarian terpenes, like β‐pinene (Singh et al., 2021). The pulp and the secondary juice (obtained after pressing the pulp from primary juice extraction), on the other hand, are important sources of carotenoids and flavonoids. Carotenoids are pigments synthesized by plants that act as precursors of vitamin A, while flavonoids are secondary plant metabolites with anti‐inflammatory, antioxidant, and anticancer properties (Russo et al., 2021). Phenolic acids (e.g., gallic, caffeic, ferulic, among others) can also be found in citrus juices and peels, with remarkable antioxidant activity (Kumar & Goel, 2019). Finally, the seeds obtained during juice extraction present large amounts of oil, proteins, and phenolic acids (Dubey et al., 2023).

**FIGURE 1:**
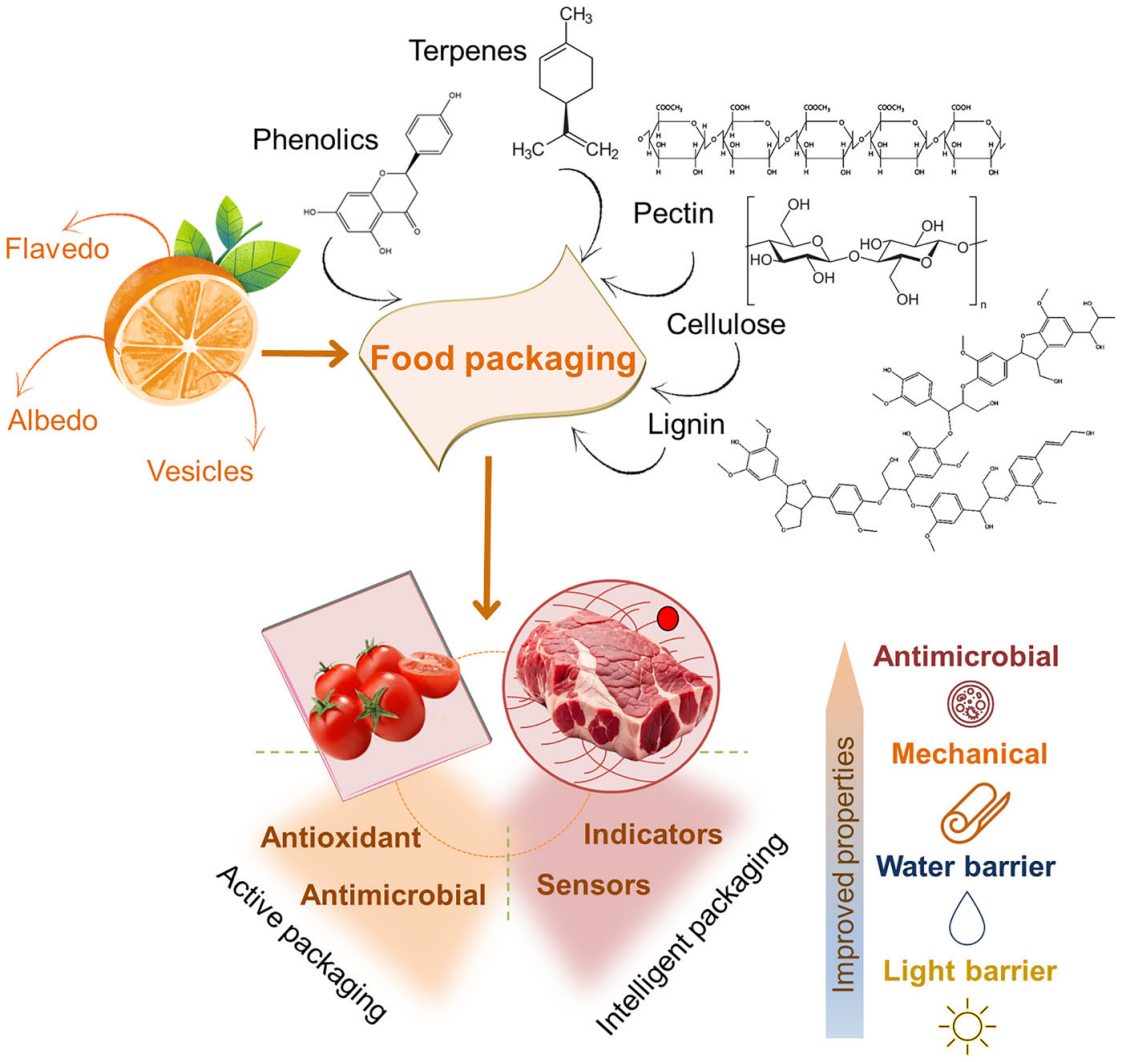
Main components of citrus peels, their primary compounds, as well as their potential applications in active and intelligent materials for food packaging.

Due to such rich composition, citrus processing waste can be reused in value‐added applications while recycling valuable compounds and preventing environmental contamination, which meets the principles of circular economy (Yun & Liu, 2022). More specifically, citrus by‐products have been used as raw materials in the preparation of food packaging films. For instance, the pectin found in these wastes can be used as the polymeric matrix for the preparation of films, and the bioactive compounds abovementioned can be added to the polymeric matrix, enhancing its functional and physical properties, including the development of active and intelligent food packaging (Otoni et al., 2021) (Figure 1).

The current scenario of citrus waste utilization in food packaging development has been recently discussed by Andrade et al. (2022), Yun and Liu (2022), and Dubey et al. (2023). Specifically, Andrade et al. (2022) highlighted some of the most used and effective citrus extraction techniques and the application of the obtained EOs and extracts directly or indirectly (through active packaging) to foods. Yun and Liu (2022) described the preparation methods, physical and functional properties, and the applications of citrus processing wastes‐based packaging films. Dubey et al. (2023) compared the application of citrus peel powder and the secondary utilization of the beneficial substances from these residues for food packaging purposes. The authors explored the role of specific compounds, such as vitamin C (ascorbic acid), as binding agents or plasticizers in packaging.

To the best of our knowledge, although previous reviews address the use of citrus waste for food packaging, none provided a detailed analysis of key physical–chemical properties of the citrus‐based food packaging materials (such as water and light barrier, tensile, morphological, among others) or how these properties can be influenced by the main compounds deriving from citrus waste. Moreover, the development of citrus‐based food packaging with intelligent properties is only briefly mentioned in the cited review papers, despite the growing importance of such materials. Thus, this review aims to provide an overview of the latest advances in the utilization of citrus waste for food packaging purposes, encouraging new research to reduce the environmental impact brought by discarded citrus by‐products while contributing to food preservation. The studies mentioned and discussed in this review were mainly published in the last 5 years (2020–2024) and are focused on developing both active and intelligent packaging films. The papers were found through the Web of Science database, searching for the words “food packaging,” “active packaging,” “intelligent packaging,” and “citrus waste” in the title, abstract, and/or keywords.

In summary, this review intends to highlight the contribution and effects of each of the citrus waste/packaging components (i.e., polymers, extracts, and EOs) on the functional and physical–chemical properties of the materials. Besides, it also discusses the main techniques employed for the extraction of phenolics and EOs, for packaging production (such as casting and electrospinning), and also for the development of citrus‐based intelligent packaging. Finally, the main gaps and possibilities for new research in the field are also discussed.

## 2 AN OVERVIEW OF INTELLIGENT AND ACTIVE POLYMER‐BASED FILMS FOR FOOD PACKAGING

**FIGURE 2:**
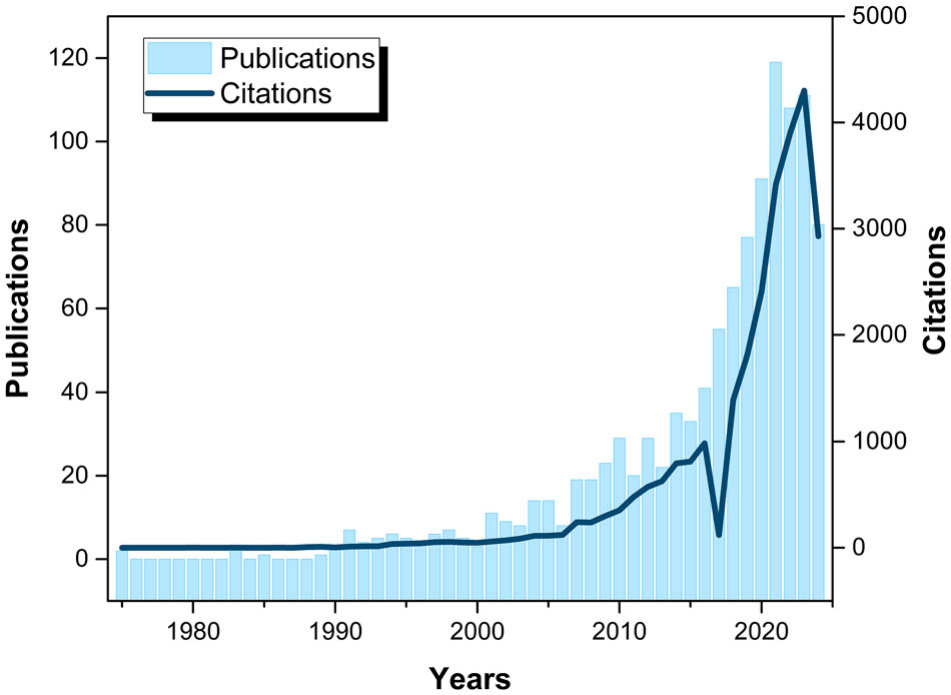
Number of publications (left side) on polymeric food packaging and average citations (right side) per year from 1975 to 2024. Data from Web of Science accessed in August 2024 using the query (ALL = (polymeric food packaging)) AND (DT = (“ARTICLE”)) (https://www.webofscience.com/wos/woscc/summary/4a239e76‐b0c4‐432f‐8d31‐1e531e6e13eb‐d53ce4c0/relevance/1).

A comprehensive search conducted in the Web of Science database yielded 1124 articles published on the development of polymeric food packing. This database records two pioneering publications from the 1970s, which focused on analyzing and simulating gas exchange in fresh foods (Hayakawa et al., 1975; Henig & Gilbert, 1975), shedding light on the variables influencing food preservation and quality in polymeric packaging. Moving into the 1980s, only five studies emerged, primarily aiming to evaluate the kinetics of chemical degradation in food, along with computer simulations of packaging decay and permeability determination (Miltz et al., 1989). The subsequent decade, spanning from 1990 to 2000, witnessed a modest increase, with no more than seven annual publications concerning polymeric packaging applied to food. However, there has been a remarkable surge in publications post‐2000s, as depicted in Figure 2, indicating an escalating interest and demand for this type of food packaging. It is noted that the state of the art in polymeric food packaging is characterized by a convergence of materials science, engineering innovations, and sustainability, all aimed at providing safe, high‐quality, and environmentally responsible packaging solutions for the modern food industry.

Packaging can be classified into two types based on its properties: intelligent packaging and active packaging. Intelligent packaging is defined as materials and devices capable of monitoring the condition of the packaged food or the environment in which it is stored (Azeredo & Correa, 2021; Cheng et al., 2022; Riahi et al., 2024). This type of system offers tools that allow real‐time, continuous, and in situ monitoring of food quality and safety (Azeredo & Correa, 2021) and usually relies on technologies based on indicators that consist of physical–chemical and electrochemical sensors or even biosensors (Cheng et al., 2022; Young et al., 2023). Among the indicators, time–temperature sensors are used to record and indicate the thermal history and the remaining shelf life of the food. Humidity sensors are used to measure the amount of water inside packages and thus indicate the quality or shelf life of the food. They are generally used for powders, cereals, and snacks packages. Many foods degrade when exposed to oxygen, so oxygen sensors are used to monitor the presence of the gas and ensure that the food has not suffered any type of oxidation that compromises its quality. pH sensors are also useful to indicate food freshness and safety, since changes in pH often occur during the degradation process of food. Microorganism sensors aim to check the presence of spoilage or pathogenic microorganisms that reduce food quality and can pose a risk to human health. Specific chemical sensors have been used to detect a series of substances that can be produced in foods unsuitable for consumption, such as toxins, biogenic amines, and sulfur compounds (Cheng et al., 2022; Young et al., 2023).

The combination of conventional sensors with food packaging requires meticulous strategies regarding safety aspects, since structural components used in electrical and optical systems can contain chemical hazards, such as dyes, conductive polymers, and heavy metals (Ghulam & Abushammala, 2023; Min et al., 2023). However, the advances in sensors’ field point to the possibility of manufacturing sensors using green‐derived natural compounds, with performance comparable with that of conventional sensors (Min et al., 2023; Teodoro et al., 2021; Xu et al., 2024). For example, natural dyes like curcumin and anthocyanins have been explored in tags for monitoring the environment inside food packaging (Carpena et al., 2023; Chen et al., 2020; Kuswandi et al., 2020). These natural dyes are mainly used in pH indicator tags but can also be applied in stimuli‐responsive systems that can trigger the release of dyes, such as by temperature (Chu et al., 2023), providing visual information about the packed food. Moreover, efforts toward the eco‐friendly synthesis of nanomaterials can also benefit the safety integration of sensors for food purposes (Keçili et al., 2025). Among these eco‐friendly routes, we can cite (i) biological synthesis using natural organisms such as bacteria, fungi, algae, and plant extracts, (ii) biomaterials from nature, such as cellulose, chitosan, lignin, and starch, (iii) physical and mechanical routes, and (iv) environmentally benign solvents, such as ionic liquids, supercritical fluids, and deep eutectic solvents (Keçili et al., 2025). All of the mentioned strategies indicate the potential of using citrus compounds for the safe application as sensor devices.

Active packaging is essential when the objective is to control bacteria and fungi that may compromise food safety. The literature defines active packaging as packaging intentionally crafted with components aimed at improving the performance of the preservation system, extending shelf life, and ensuring food quality (Firouz et al., 2021; Riahi et al., 2024; Young et al., 2023). These components may reside within the packaging material or in the available space. Consequently, the prolonged product usability correlates with its interaction with the packaging or the enclosed airspace. Food quality is maintained through physical, chemical, or biological reactions facilitated by active packaging. To accomplish this, active packaging employs various strategies, including oxygen scavengers, ethylene absorbers, carbon dioxide absorbers/emitters, flavor release/absorption systems, antioxidants, antimicrobials, and humidity controllers (Azeredo & Correa, 2021). In this review, the antioxidant and antibacterial functions are highlighted. The use of active packaging with antioxidant functionality aims to reduce the oxidation of food components. Therefore, such systems can help to preserve food quality over an extended period. This strategy can be implemented through the release of antioxidant substances into the food environment or by applying a coating to the food surface (Azeredo & Correa, 2021; Firouz et al., 2021; Yadav et al., 2023).

The main differences between active and intelligent packaging lie in their functions and capabilities. While active packaging directly influences the product or its environment to preserve or enhance food quality, intelligent packaging aims to monitor or display information about the product quality status without direct interaction with it. However, it is possible to achieve both functions by combining these characteristics. For example, Guo et al. (2021) developed an active–intelligent film based on pectin extracted from watermelon, incorporating beetroot extract. The beetroot extract served both to indicate changes in the food pH and to reduce oxygen and water vapor permeability (WVP) (Guo et al., 2021). Similarly, Jiang et al. (2023) proposed packaging for shrimp utilizing films composed of pectin and betacyanins extracted from pitaya peel waste. This material responded to pH variations, while the betacyanins enhanced UVbarrier properties and antioxidant capabilities of the pectin films (Jiang et al., 2023).

## 3 POLYMERS OBTAINED FROM CITRUS PROCESSING WASTE

The chemical composition of peel, pomace, and seeds of citrus byproducts depends on the climatic conditions to which the plant is exposed (Yadav et al., 2023). Peels are rich in sugars and EOs (in the flavedo) and present a high content of pectin (in the albedo), while seeds contain nitrogen‐free extracts, lipids, crude proteins, and fibers (Andrade et al., 2022). Pomaces are rich in fibers, sugars, polysaccharides, phytochemicals, phenolic compounds, natural antioxidants, and other nutrients (Maqbool et al., 2023). Therefore, these three types of citrus waste are potential natural sources of biopolymers and other molecules of interest for the food packaging industry, as indicated in Figure 1. Table 1 describes the content of polymers in general citrus waste (dry powder bagasse), peels, pomace, and seeds. Peels and dry powder bagasse of general waste are the major explored by‐products for pectin and cellulose extraction, and while these polysaccharides are the main biopolymers found in citrus waste, hemicellulose and lignin are also found and can be extracted for packaging applications.

**TABLE 1:** Main citrus polymers and their contents according to the type of citrus waste.

Type of waste	Source	Citrus polymer	Content (%)	Reference
Peels	Citrange (albedos)	Pectin	29.0	Chandel et al., 2022
	Lime	Pectin	5.2–23.6	Chandel et al., 2022
	Grapefruit	Pectin	23.4–26.7	Chandel et al., 2022
	Lemon	Pectin	13.0	Magalhães et al., 2023
		Cellulose	23.1	
		Hemicellulose	8.1	
		Lignin	7.6	
	Mandarin	Cellulose	22.5	Dubey et al., 2023
		Hemicellulose	6	
		Lignin	10	
	Orange	Cellulose	12	de Castro et al., 2024
		Pectin (galacturonic acid)	88	
		Lignin	2.2	
	Orange	Hemicellulose	11	Dubey et al., 2023
Pomaces	Citrus in general	Pectin	14–25	Maqbool et al., 2023
	Citrus in general	Hemicellulose + cellulose	13–17	Maqbool et al., 2023
	Lemon	Pectin	22.5	Magalhães et al., 2023
		Cellulose	36.2	
		Hemicellulose	11.1	
		Lignin	7.6	
Seeds	Lemon	Cellulose	19.2	Zhang et al., 2020
		Hemicellulose	13.3	
		Lignin	9.1	
General bagasse (powder)	Orange	Cellulose	12.4	Mantovan et al., 2023
		Hemicellulose	7.5	
		Lignin	8.9	
	Lime (without seeds)	Cellulose	8.9	Impoolsup et al., 2020
		Hemicellulose	1.4	
		Lignin	10.1	

Except for lignin, citrus polymers are mostly carbohydrate polymers. Carbohydrate polymers can be classified according to their function as storage polysaccharides (e.g., starches), structural polysaccharides (e.g., cellulose and pectin), and gel‐forming polysaccharides (e.g., alginate and pectin) (Correa et al., 2024). These polymers are present in vegetal cell walls and middle lamella, building a hierarchical structure in which cellulose microfibrils are wrapped and cross‐linked with amorphous matrices of hemicellulose and lignin and a gel‐like subnetwork of pectins, beside other compounds. The synthesis of these different polysaccharides obeys a sophisticated and well‐coordinated biosynthetic mechanism orchestrated by specialized enzymes called glycosyltransferases, which link the different sugar moieties in unique designs (Held et al., 2015).

Pectin is one of the main components of fruits and vegetables, and citrus residues are its majority source, second only to apples (Maqbool et al., 2023; Yadav et al., 2023). Chemically, pectin is composed of chains of 300–1000 galacturonic acid units bonded by α‐(1→ 4) glycosidic linkages (Figure S1A), which results in a water‐soluble anionic polymer (Chandel et al., 2022; Mellinas et al., 2020). Nonetheless, pectins can vary according to their molecular weight and chemical configuration, resulting in polymers with diverse functional properties (Mellinas et al., 2020). Although the generic structure of a linear polygalacturonic acid (called homogalacturonan) corresponds to about 60% of the pectin in cell walls, more complex molecular structures are also attributed to pectin, like the rhamnogalacturonan I and rhamnogalacturonan II, whose backbones contain other sugars and ramifications (Chandel et al., 2022; Nastasi et al., 2022).

Cellulose is the second most abundant citrus polymer and, from a wider perspective, the most abundant organic compound on Earth (Andrade et al., 2022). Its chemical structure comprises linear polymer chains of β‐(1→ 4) glycosidic linkages, as shown in Figure S1B (Correa et al., 2024). The high number of hydroxyl groups along these linear polymer chains determines the physical–chemical properties of cellulose, given by the cohesiveness arising from the strong intra‐ and intermolecular hydrogen bonds (Teodoro et al., 2021). Cellulose is extracted as short fibers, and the interest in this polymer has been expanded as a result of the advances related to the remarkable properties of its nanosized structures (called nanocelluloses or cellulose nanomaterials) (Teodoro et al., 2021b). The International Organization for Standardization (ISO/TS 20477:201748) classified the nanocelluloses as bacterial cellulose (BC), cellulose nanofibrils/microfibrils (CNFs or CMFs), and cellulose nanocrystals (CNCs), classification and nomenclature also adopted in this review (Teodoro et al., 2021b).

Along with cellulose, hemicellulose and lignin represent the bulk of insoluble fibers of citrus wastes (Maqbool et al., 2023; Russo et al., 2021). The hemicelluloses belong to a group of heterogeneous sugars, whose molecular chains are constituted of pyranose and furanose units, including d‐xylose, d‐mannose, d‐glucose, d‐galactose, l‐arabinose (Figure S1C), among others (Rao et al., 2023). As aforementioned, despite being one of the most abundant polymers in citrus waste, lignin is not classified as a polysaccharide. Unlike other abundant citrus polymers, it presents a complex and amorphous structure of polyphenols, as exemplified by a general chemical model in Figure S1D. Its chemical composition includes three main units—syringyl, guaiacyl, and p‐hydroxyphenyl—resulting from the phenolics sinapyl, coniferyl, and p‐coumaryl alcohols (Sharma et al., 2023). The number of methoxy groups and the distribution of the monomeric units on the final lignin structure depend on the source of biomass and the chemical procedures used for its isolation (Sharma et al., 2023).

The wide availability of citrus biopolymers and their intrinsic nontoxicity and biodegradability have led to their market share in the plastics industry (Mellinas et al., 2020). However, transposing biopolymers from their natural functions to replace conventional packaging requires several studies to optimally benefit from their properties and meet packaging requirements. Citrus waste‐based packaging is expected to be able to protect food from the external environment (Dubey et al., 2023) and present appropriate mechanical resistance and flexibility (Dubey et al., 2023), as well as transparency and light transmission ability (Khalid et al., 2024; Kim et al., 2024), gas barrier properties (against oxygen, carbon dioxide, and ethylene), antimicrobial effects, and antioxidant activity (Andrade et al., 2022).

**FIGURE 3:**
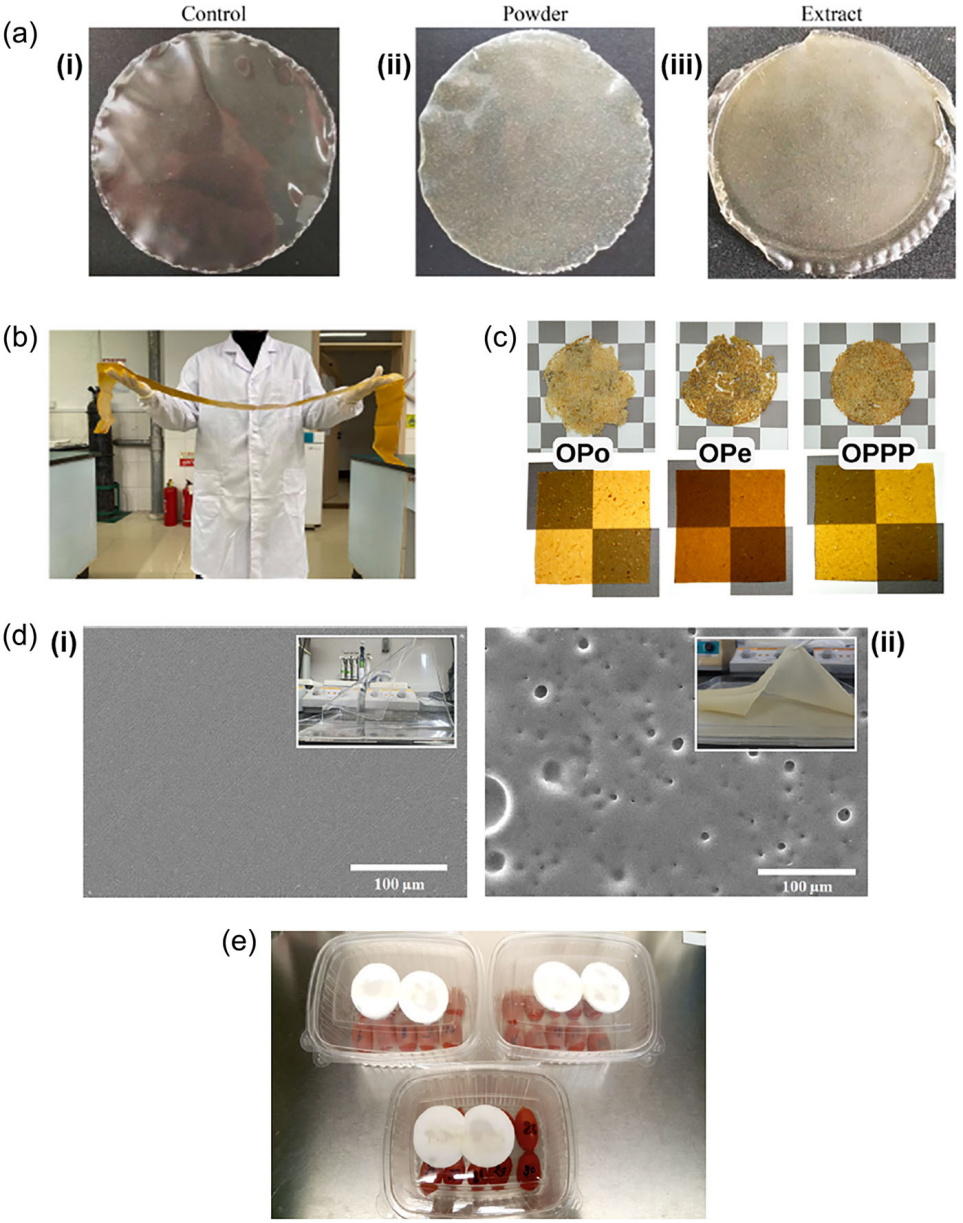
Examples of food packaging materials based on citrus byproducts: (a) (i) cassava starch‐based films (control) containing (ii) orange residue in the powder form and (iii) orange residue in the aqueous extract form (Claudia‐Leites et al., 2021) (reprinted with permission of Elsevier, 2024); (b) semitranslucent pectocellulosic bioplastics with excellent mechanical properties (Zhang, Fu, et al., 2023) (reprinted with permission of Elsevier, 2024); (c) films obtained from orange pomace (OPo), orange peel (OPe), and mixed pomace, peel, and finisher pulp (OPPP) (Santos et al., 2023) (reprinted with permission of Elsevier, 2024); (d) SEM images of (i) bioelastomers of polydimethylsiloxane (PDMS) and (ii) bioelastomers with mandarin peel extracts (Lee al. et al., 2023) (reprinted with permission of MDPI, 2024); and (e) application of films of grapefruit peel pectin containing grapefruit peel extract as antimicrobial packaging in the storage of tomatoes (Khalil et al., 2022) (reprinted with permission of Elsevier, 2024).

Polysaccharides exhibit a filmogenic nature, meaning that strong films can be formed when these materials are in solid state, which is directly related to intermolecular cohesion (Kocira et al., 2021). Moreover, the inclusion of citrus polymers in packaging formulation is facilitated due to their good compatibility with other compounds, such as synthetic biopolymers, proteins, and lipids (Kim et al., 2024). They can be included for strategic functions, assisting in the rheological control of film‐forming solutions, acting as thickening and/or gelling agents or texturizers, aiding the encapsulation of substances for Pickering emulsions, and serving as colloidal stabilizers (Mellinas et al., 2020). In this sense, a myriad of investigations has demonstrated the successful fabrication of films for packaging using a matrix composed of citrus‐based polysaccharides and lignin (Li et al., 2024; Yun & Liu, 2022), or where these polymers are applied as additives to provide specific properties to the packaging, as illustrated in Figure 3 (Kim et al., 2024).

Figure 3a shows cassava starch‐based films incorporated with orange residues to improve their antioxidant and antimicrobial functions: (i) the control films, (ii) the films containing orange residue in the powder form, and (iii) the ones with orange residue in the aqueous form (Claudia‐Leites et al., 2021). Figure 3b shows flexible and semitranslucent bioplastic films prepared on a large scale with pectin and cellulose (Zhang, Fu, et al., 2023). Films entirely based on orange pomace (OPo), orange peel (OPe), and mixed pomace, peel, and finisher pulp (OPPP) are shown in Figure 3c (Santos et al., 2023). Figure 3d shows scanning electron microscopy (SEM) images of (i) bioelastomers of polydimethylsiloxane (PDMS) and (ii) bioelastomers with mandarin peel extracts (Lee al. et al., 2023). Finally, Figure 3e presents the application of films of grapefruit peel pectin containing grapefruit peel extract as antimicrobial packaging in the storage of tomatoes (Khalil et al., 2022).

More specifically, since pectin represents the soluble dietary fibers of citrus waste, it has been widely explored for packaging films and coatings (Nastasi et al., 2022). Cellulose and its nanostructures have also been applied as natural mechanical reinforcement agents (Yadav et al., 2023); in contrast, due to the branching and amorphous chemical structure of hemicelluloses, their films lack long‐term thermal stability and mechanical strength (Li et al., 2024). However, the inclusion of hemicelluloses in packaging formulation is convenient due to their contribution to gas barrier property improvements, such as the preferable oxygen and water barrier properties exhibited by blends of cellulose and hemicelluloses (Dubey et al., 2023; Li et al., 2024). Regarding optical properties, transparent and semitransparent films can be obtained using pectin (Mellinas et al., 2020), cellulose, and hemicellulose (Kim et al., 2024). The hydrophilicity of packaging can be reduced by using lignin, and additional properties for active packaging can be achieved using this polymer. Due to its complex phenolic structure (presenting functional groups such as phenolic units, ketones, and chromophores), lignin can provide UV‐blocking properties, as well as antimicrobial and antioxidant properties (Khalid et al., 2024).

One of the most employed methods for producing packaging films is the casting technique. Its widespread adoption arises from its simplicity and accessibility, since it does not demand any specific or specialized equipment (Roy et al., 2023). The method consists of three main steps: dissolving, casting, and drying. First, a suitable solvent is chosen to dissolve the components of the film. In this regard, pectin and cellulose enable using water as a solvent, which is a remarkable advantage. Plasticizers and other additives may also be used to produce films with desired mechanical and physical–chemical properties. For films designed to be in contact with food, the components must be nontoxic, and contamination should be strictly avoided (Roy et al., 2023). Heat is frequently used to enhance and speed up the dissolving process (Prakoso et al., 2023).

Before casting, occasional bubbles can be removed to produce a smoother surface. Then, the solution is cast into a predefined mold. The casting approach allows producing large‐size films in predefined formats. Furthermore, the technique allows the film to be deposited onto flexible and nonporous substrates. Lastly, the drying process consists of solvent evaporation. Proper definition of drying conditions is critical, as this step significantly influences the properties of the final film. Typically, temperatures slightly above room temperature (<50°C) are employed. However, a drawback of this process is the extended drying time (ranging from several hours to a few days), which may hinder its commercial viability (Roy et al., 2023).

Mousavi Kalajahi et al. (2022) used orange waste powder to produce a biocomposite film by casting. Taking advantage of the presence of pectin in the waste powder, CNF was used as a structural reinforcement and nettle EO as an antibacterial agent. The films were obtained by casting the prepared solutions on polystyrene plates and drying them at 35°C for 36 h. The film fabrication method enabled the interaction of CNF with pectin, which led to reduced WVP and improved physical properties. The blending ratio of pectin and pullulan for food packaging was investigated by Priyadarshi et al. (2021). The simplicity of producing the films by casting the solution onto Teflon‐coated glass plates enabled the investigation of five different film compositions. The interaction of the components led to H‐bonds that enhanced the strength and stiffness of the films. The 50:50 ratio exhibited the lowest oil and water absorption values.

## 4 EXTRACTS OBTAINED FROM CITRUS PROCESSING WASTE

Besides carbohydrate polymers and lignin, citrus residues are also a valuable source of phenolic compounds, such as phenolic acids (caffeic, p‐coumaric, ferulic, and sinapic acids) and flavonoids (flavanones, flavones, isoflavones, flavanols, and anthocyanins). These molecules are found in peels, pressed pulp, pomace, seeds, and/or leaves and can be used to protect cells against the action of free radicals due to their strong antioxidant property. Moreover, they also have antibacterial, antiviral, anti‐inflammatory, anticancer, and antiallergic action, in addition to protecting against chronic diseases (Singh et al., 2020). The largest amount of phenolic compounds is found in the peels (Sorrenti et al., 2023), and flavonoids can be mostly found in both the peels and pulp of citrus wastes. The main phenolic compounds found in citrus peels, as well as their structural chemical formulas, are presented in Table S1.

Different extraction methods can be used to process citrus waste in order to recover their phenolic compounds and take advantage of their aforementioned properties, providing added value to what would otherwise be discarded (Nirmal et al., 2023). The extracts obtained after extraction, whether in powder form or in an aqueous medium, have been widely used as additive agents in the production of films or packaging for the food sector (Khalil et al., 2022; Merino et al., 2023; Santos et al., 2023; Shen et al., 2024; Xu, Ding, et al., 2024; Yun et al., 2023; Zhang, Fu, et al., 2023).

The phenolics extraction methods can be classified as conventional (solid–liquid extraction, such as maceration, percolation, decoction, reflux, and Soxhlet) or nonconventional (such as supercritical fluid extraction [SFE], microwave‐assisted extraction [MAE], enzyme‐assisted extraction, pressurized hot water extraction, ultrasound‐assisted extraction [UAE], and pulsed electric field‐assisted extraction) (Nirmal et al., 2023). These methods can use different strategies (grinding, heating, solvents, drying, particle size classification, biochemical processes, and equipment) to process and classify citrus wastes to obtain extracts with a preserved phenolic profile that are at the same time economically viable (Nirmal et al., 2023). In conventional methods, the physical and chemical properties of the solvents used, as well as the temperature applied during extraction, can directly influence the extraction efficiency (Nirmal et al., 2023). Despite being widely used for the extraction of phenolic compounds from citrus waste, these conventional methods present some drawbacks, such as high costs, usage of flammable solvents, long extraction times, low extraction efficiency, and low purity of the obtained extracts (Stabrauskiene et al., 2022). Thus, to overcome these disadvantages, novel and more efficient extraction methods based on environmentally friendly technologies have been pursued. These nonconventional methods consist of modern extraction technologies that are more efficient, cost‐effective, and expeditious (requiring shorter extraction time), besides being environmentally viable (Rifna et al., 2023).

SFE is a cutting‐edge technique that uses supercritical carbon dioxide (CO_2_) as a solvent to extract phenolic compounds from citrus peels. CO_2_ has several advantages: it is economical, easily available in a pure state, and safe to use. Supercritical fluids have a density similar to that of a liquid, giving them excellent solvation power. Furthermore, their relatively low viscosity and high diffusivity improve mass transfer, reducing extraction time. The application of lower temperatures during the process preserves heat‐sensitive compounds, ensuring the quality and functionality of the products obtained. However, SFE presents some limitations, such as the lack of selectivity for polar compounds. Moreover, the need to monitor temperature and pressure requires a significant investment in equipment (Picot‐Allain et al., 2021).

In the case of MAE, nonionizing radiation in the form of microwaves is applied, with frequencies between 300 MHz and 300 GHz. Microwaves initiate ionic conduction, generating an electric field that causes dielectric heating. This leads to the rotation of polar molecules, releasing energy as heat. The increase in the pressure inside the plant cells due to the evaporation of moisture causes swelling and rupture, exposing the cells to the solvent. It is also possible to apply this system without solvents, using the water present in the sample as a solvent. It is effective for extracting bioactive compounds from plants and is faster than other methods such as supercritical fluid and pressurized liquid extraction (Picot‐Allain et al., 2021). Some processing parameters need to be considered during MAE, such as microwave generation power, extraction time, type, size, and moisture content of the sample, as well as the type of solvent and organic additives used.

UAE consists in the removal of bioactive compounds by generating mechanical waves at frequencies from 20 to 100 kHz, which promotes cavitation process (Li, Lei, et al., 2021), being a versatile, environmentally friendly, low‐cost, and easy‐to‐operate method. Like MAE, some parameters need to be controlled during UAE for phenolics recovery, such as temperature, sonication time, work frequency, and sample size (Rifna et al., 2023). The pressurized hot water extraction method, in turn, is based on applying constant pressure and temperature values in the range from 100 to 374°C. This method employs heated water as a solvent, thus allowing the extraction of less polar chemicals with high efficiency (Leo & Ong, 2024).

The pulsed electric field‐assisted extraction method is based on the application of a pulsed electric field with an intensity from 0.1 to 100 kV cm^−1^ that promotes cellular electroporation of food, being used in peeling, cooking, extraction, drying, and freezing‐thawing of citrus waste in the solid state (Zhang, Lyu, et al., 2023). Furthermore, it allows a shorter processing time, lower energy consumption, lower temperature variation during extraction, and greater mass transfer, and it is also an environmentally friendly method. Nevertheless, some challenges still need to be overcome (like the generation of a stable and continuous high‐intensity electric field and the development of novel electrodes to improve the useful life of these devices) so that this method can be widely used in the food industry (Zhang, Lyu, et al., 2023).

Finally, the enzyme extraction method consists of using enzymes such as proteases, cellulase, amylase, glucosidase, pectinase, and hemicellulase to extract phenolic compounds, EOs, carotenes, and terpenes from citrus residues with their biological, bioactive, and physical–chemical properties preserved (Gligor et al., 2019). This method allows the extraction of components present in the plant cell wall, has high extraction efficiency due to the high enzymatic specificity, is used in the extraction of heat‐sensitive waste, and is also an environmentally friendly method. However, it requires high investment, and few enzymes have been studied and optimized regarding their extraction efficiency so far (Gligor et al., 2019).

Table 2 highlights the most used citrus waste materials employed for the extraction of phenolic compounds for the production of active and intelligent packaging. Moreover, it highlights the type of processing and extraction of the wastes, the concentration of the extracts added to the packaging, as well as the type of packaging developed in each study. The data presented in Table 2 show that, although the conventional methods are still widely used for the extraction of phenolic compounds from citrus wastes, the nonconventional methods like MAE (Lee et al., 2023; Singhi et al., 2023) and UAE (Tone et al., 2024) have gained prominence lately. However, these new methods still need to overcome some scientific and technological challenges to meet the demands of implementation, logistics, purchase of equipment, operational costs, and training of qualified labor, in order to be widely used by the food industry.

**TABLE 2:** Main citrus waste sources used for the extraction of phenolic compounds and essential oils for the production of active food packaging.

Citrus waste source	Citrus waste processing/extraction method	Concentration of phenolic extracts used in the fabrication of the packaging	Application	Reference
Orange (*Citrus sinensis*) peels and bagasse	Grinding and soaking the powder in water, followed by filtration	10% (w/w) for the powder and replacement of water by the aqueous extract	Active packaging film with antibacterial performance in vitro	Claudia Leites et al., 2021
Orange (*Citrus sinensis*) peels	Freeze‐drying, hammer mill, sieving	46% (w/w)	Active packaging film with antifungal performance in vitro	McKay et al., 2021
Bitter orange (*Citrus aurantium* L.) peel	SFE (CO_2_ and ethanol)	0.67%, 0.75%, 0.96%, 1.11%, 1.32%, 1.56%, and 1.71% (w/w)	Active packaging films with antibacterial and antioxidant performance in vitro	Casas Cardoso et al., 2022
Grapefruit (*Citrus paradisi*) peel and lemon (*Citrus lemon*) peel	Freeze‐drying, grinding, sieving, encapsulation	0.04% (w/w)	Active edible film with antibacterial and antioxidant performance in vitro and antimicrobial performance in the wrapping of cherry tomatoes	Khalil et al., 2022
Orange (*Citrus sinensis*) peel flour	Grinding and heating	0%–40% (w/w)	Active packaging films with antioxidant performance in vitro	Barone et al., 2023
Mandarin (*Citrus reticulata*) peel	MAE	0%–20% (w/w)	Active bioelastomer for food packaging with antibacterial and antioxidant performance in vitro	Lee et al., 2023
Sweet lemon (*Citrus limetta*) pomace	MAE and grinding	5%, 10%, and 20% (v/v)	Active packaging films with antioxidant performance in vitro	Singhi et al., 2023
Orange (*Citrus sinensis*) peel	UAE and solvent (ethanol and water) extraction	7.5% and 10% (w/w)	Active packaging films with antibacterial and antioxidant performance in vitro	Tone et al., 2024
Lime (*Citrus aurantium* L.) drop	Sieving, heating, centrifugation, evaporation, and freeze‐drying	1%, 3%, 5%, and 10% (w/w)	Active edible films with antioxidant performance in vitro and applied as strawberry coatings	Xu et al., 2024

Citrus waste source	Method of EOs obtention	Concentration of EOs used in the fabrication of the packaging	Application	Reference
Rangpur lime (*Citrus limonia*) leaves	Hydrodistillation (2 h of boiling)	0.4%–2% (v/v)	Active packaging film with antibacterial performance in vitro	de Oliveira Filho et al., 2020
Sweet orange (*Citrus sinensis*) peel	Direct distillation (100°C, 3 h extraction time)	2.5% and 5% (v/v)	Active packaging film with antibacterial and antioxidant performance in vitro	Amjadi et al., 2021
Sweet lime (*Citrus Limetta*) peel	Vacuum‐assisted solvent free microwave extraction (800 W microwave output power and 0.5 h extraction time)	1%–3% (v/v)	Active packaging films with antibacterial performance in vitro and applied as fish fillet wrappers	Arafat et al., 2021
Mandarin (*Citrus reticulate* L.) peel	Hydrodistillation (2 h)	10% (v/v)	Active packaging electrospun webs with antibacterial performance in vitro	Terzioğlu et al., 2022
Bergamot (*Citrus bergamia*)	Cold pressing	0.1% and 0.2% (v/v)	Active edible films with antibacterial performance when applied as strawberry coatings	Bruno et al., 2023
Grapefruit (*Citrus paradisi*)	Distillation (100°C)	0.5%–2% (v/v)	Active edible film with antibacterial performance when applied as lamb slices coatings	Zanganeh et al., 2023

*Note*: Data refer to the last 5 years (2020–2024). The essential oils shown in the table were obtained from citrus wastes and not purchased commercially.

Abbreviations: MAE, microwave‐assisted extraction; SFE, supercritical fluid extraction; UAE, ultrasound‐assisted extraction.

*Citrus sinensis*, or sweet orange, is the most applied citrus in the studies presented in Table 2, and its peels were the most evaluated waste source (Barone et al., 2023; Claudia Leites et al., 2021; McKay et al., 2021; Tone et al., 2024). Other citrus like mandarin, lemon, and grapefruit were studied by different authors (Khalil et al., 2022; Lee et al., 2023; Singhi et al., 2023). Moreover, citrus bagasse and pomace were also applied as sources of phenolic compounds. All the studies applied the citrus extracts obtained as additive agents in polymeric matrixes, aiming to develop active food packaging materials (mostly films). The extracts were incorporated into the films in different ways and at different concentrations, depending on the method of incorporation (i.e., added at a certain concentration in relation to the mass of the polymer used, when in powder, or solubilized at a certain concentration in the ethanolic solutions). The incorporation of these extracts certainly led to changes in the mechanical, thermal, antioxidant, antimicrobial, optical, and rheological properties of the films, as well as in their permeability to the passage of water and gases, among others. These properties and how the citrus extracts addition affected them will be discussed in detail in Section 6.

## 5 EOs OBTAINED FROM CITRUS PROCESSING WASTE

Citrus wastes can also be an important source of EOs, which are complex mixtures of volatile substances with concentrations ranging from 85% to 99%, comprising around 400 compounds, which may or may not be water soluble (Khalid et al., 2024). These compounds are stored in small sacks or glands distributed throughout several layers of the citrus peel, with a greater concentration in the region known as flavedo. The main constituents of citrus EOs include monoterpene hydrocarbons, sesquiterpene hydrocarbons, oxygenated monoterpenes, and oxygenated sesquiterpenes (Singh et al., 2021). Limonene, a monoterpenic hydrocarbon, stands out as the main component of citrus EOs, representing from 32% to 98% of their total composition; other commonly found bioactive terpenes are mirsen, β‐myrcene, α‐pinene, sabinene, β‐pinene, α‐terpinene, linalool, tocopherols, sitosterol, α‐tocopherol, catechin, octanal, decanal, among others (Khalid et al., 2024). The content and composition of EOs may vary from one citrus species to another, with concentrations between 0.5% and 5.0% (Singh et al., 2021). Environmental factors such as climate, altitude, time of harvest, and degree of ripeness of the fruits also have great influence, along with the extraction and separation methods used (Paw et al., 2020).

As in the case of phenolic compounds, EOs from citrus waste can be obtained through different routes; the conventional methods include cold pressing, solid–liquid extraction, liquid–liquid extraction, steam distillation, infusion, and Soxhlet extraction (Grover et al., 2024). Hydrodistillation, the oldest and simplest method for extracting EOs, is employed to obtain oils from a variety of sources, including flowers, fruits, leaves, and seeds. However, the interaction of water with biomass during this process can lead to hydrolysis and the formation of alcohol and carboxylic acids, potentially altering the EO composition (Sulzbach et al., 2021).

Steam distillation is another traditional method widely used to obtain citrus EOs; it involves passing steam through the citrus peel material, which vaporizes volatile compounds. The subsequent condensation of this vapor leads to the separation of the EO and the water. It is a simple and economical process, but it can degrade some heat‐sensitive compounds, in addition to not separating nonvolatile compounds and being a time‐demanding process (Lubis et al., 2023). Cold pressing is one of the most employed processes in the extraction of EOs. In this method, citrus peels are mechanically pressed to release the EOs present in the peel glands, forming an aqueous emulsion without the application of heat; this emulsion is then centrifuged to recover the EOs. This process is valued for preserving the integrity of heat‐sensitive compounds. However, it may not extract EO as efficiently as steam distillation and may require larger quantities of raw materials to produce the same volume of oil (Singh et al., 2021).

New EO extraction methodologies have emerged to overcome the challenges of the abovementioned conventional processes, minimizing process losses, increasing efficiency, reducing extraction time, and minimizing changes in the composition of the EOs. Some of these techniques are the same as those outlined in the previous section for phenolics extraction (e.g., SFE, MAE, and UAE) and accelerated solvent extraction (ASE). Regardless of how they are obtained, citrus EOs have proven to be excellent natural substitutes for synthetic substances commonly used in packaging due to their antimicrobial and antioxidant properties. Incorporating these oils into packaging materials can help to preserve the freshness and integrity of food products, minimizing waste, increasing their stability during storage, and allowing the disposal of the packaging without environmental impacts.

EOs can be incorporated into different polymeric matrices, natural or not, such as pectin, collagen, gelatin, chitosan, poly(lactic acid) (PLA), starch, poly(butylene adipate‐co‐terephthalate) (PBAT), carrageenan, alginate, and blends (Yun & Liu, 2022). Packaging materials incorporated with EOs can be produced as films, coatings, or mats (Arafat et al., 2021; Bruno et al., 2023; Terzioğlu et al., 2022). Incorporating EOs into these matrices not only alters their chemical properties but also affects their physical characteristics. Typically, this results in decreased transparency and tensile strength (TS), while elongation at break tends to increase (Li, Tang, et al., 2021). These properties and how the EOs addition affected them are discussed in Section 6.

Table 2 presents EOs that were extracted from various citrus sources, like sweet lime (*Citrus limetta*), rangpur lime (*Citrus limonia*), bergamot (*Citrus bergamia*), sweet orange (*Citrus sinensis*), mandarin (*Citrus reticulate* L.), and grapefruit (*Citrus paradisi*), and how they were obtained and incorporated into different films for active packaging. Similar to phenolic compounds, a variety of extraction methods have been used to obtain EOs. These methods range from traditional techniques such as direct distillation (Amjadi et al., 2021; Zanganeh et al., 2023) and hydrodistillation (Terzioğlu et al., 2022) to more innovative approaches like vacuum‐assisted solvent‐free microwave extraction (Arafat et al., 2021). All of the EOs from Table 2 were obtained aiming at antioxidant and/or antimicrobial properties for incorporation in active food packaging, with concentrations ranging from 0.1% to 10% (v/v).

Among the studies presented in Table 2, Arafat et al. developed a film of sweet lime (*Citrus limetta*) peel residue, in which 3% (v/v) of sweet lime EO was incorporated as an active agent; the films showed enhanced resistance to microbial proliferation during the 12‐day storage of fish fillets, when compared to a polyethylene packaging (Arafat et al., 2021). Although EOs have been demonstrated as an alternative to chemical preservatives in active food packaging, their use may be limited due to their high volatility, low aqueous solubility, and propensity for chemical degradation (de Souza et al., 2021). Therefore, encapsulation has been proposed as a key approach to maximize the efficiency of EOs. In this direction, Amjadi et al. introduced a novel approach utilizing a nanoemulsion of sweet orange (*Citrus Sinensis*) EO incorporated into whey protein isolate films, which have antimicrobial and antioxidant properties. Their study compared this nanoemulsion with an emulsion containing the same EO. The nanoemulsion significantly improved the water barrier properties of the film samples; there was also an improvement in the antioxidant and antimicrobial properties for the system that employed nanostructures compared to the traditional emulsion (Amjadi et al., 2021).

Among the various encapsulation approaches, electrospinning is a simple but versatile and facile technique to prepare nanostructured materials with a relatively high manufacturing rate and low cost (Wang & Su, 2024). Electrospinning is a fiber manufacturing technique that emits charged jets under the action of an external electric field to produce nonwoven fibers with nano‐ or micrometer diameters. The electrospun materials can be produced with the desired properties by optimizing the electrospinning parameters and the solution. Owing to their high surface‐to‐volume ratio, high porosity, and tunable properties, electrospun materials can offer many benefits in food packaging, including high encapsulation efficiency, allowing protection of the bioactive substances and compounds from external agents, and enabling controlled release at the target food (Schmatz et al., 2019). As an example, lemon myrtle essential oil (LMEO) was incorporated into cellulose acetate (CA) electrospun nanofibers (Beikzadeh et al., 2020). The LMEO‐loaded CA showed sustained LMEO release over a prolonged time and excellent antimicrobial activity against *Staphylococcus aureus* and *Escherichia coli* even after 2 months of storage. In another work, PLA nanofibers loaded with limonene oil were effective in inhibiting the proliferation of three different species of bacteria, that is, *E. coli*, *S. aureus*, and *Bacillus subtilis*, over a period of 4 weeks (Williams et al., 2024). Recently, good antimicrobial and antioxidant activities were observed after the incorporation of bitter OPe EO (up to 100 µL mL^−1^) in Eudragit/collagen nanofibers (Talib Al‐Sudani et al., 2024). The electrospun mat effectively inhibited the growth of *E. coli* and *S. aureus* while scavenging up to 50% of 2,2‐diphenyl‐1‐picrylhydrazyl (DPPH) radicals.

## 6 PHYSICAL–CHEMICAL PROPERTIES OF INTELLIGENT AND ACTIVE FILMS BASED ON CITRUS WASTE

When developing new food packaging materials, it is crucial to assess their key physical–chemical, morphological, and thermal properties. Additionally, it is important to characterize these materials according to their application (whether as active or intelligent packaging). Table 3 highlights studies of the last 5 years (2020–2024) that developed food packaging materials using citrus biopolymers, citrus phenolic extracts, and/or citrus EOs, along with their main barrier, tensile, morphological, thermal, active, and intelligent properties. The main properties and effects observed are discussed in detail in this section.

**TABLE 3:** Food packaging materials developed using citrus biopolymers, citrus phenolic extracts, and/or citrus essential oils reported in the last 5 years (2020–2024) and their main barrier, tensile, morphological, thermal, active, and intelligent properties.

Material	Water‐related properties	Gas barrier properties	Light‐barrier and optical properties	Tensile properties	Morphological, thermal, and rheological properties	Biodegradability properties	Antioxidant, antimicrobial, and intelligent properties	Reference
Mandarin (*Citrus unshiu*) peel pectin films containing sage (*Salvia officinalis*) leaf extract (0.6%, 1.0%, and 1.4% w/v)	Increase in the WVP and water solubility with higher extract concentrations	–	Increase in the yellowness and opacity with higher extract concentrations	Decrease in the TS values with 1.0% and 1.4% (w/v) of extract	Extract addition led to less homogeneous microstructures; slight decrease in the thermal stability with higher extract concentrations	–	Increase in the antioxidant activity with higher extract concentrations	Han & Song, 2020
Cassava starch films with orange peel powder (OPP) or aqueous extract	Increase in the solubility for the films containing the aqueous extract; no changes in WVP	–	Decrease in the transparency for the films containing the powder residue	Increase in the TS and Young's modulus values for the films containing the powder residue; decrease in the TS and Young's modulus values for the films containing the aqueous extract	Decrease in the homogeneity for the films containing the powder residue; decrease in the viscosity for both additions, mainly for the films with the aqueous extract	Acceleration in the degradation for the films containing the aqueous extract	Low antimicrobial activity against *Staphylococcus aureus* (inhibition zone) for both films	Claudia Leites et al., 2021
Linear low‐density polyethylene (LLDPE) antimicrobial films with OPP	Increase in the WVP with OPP addition (but still lower than other common plastics)	Decrease in the oxygen permeability with OPP addition	Decrease in the brightness and increase in the yellowness/orangeness with OPP addition; increase in the UV barrier properties (zero transmittance at wavelengths of 280 and 320 nm)	Decrease in the TS and EB values with OPP addition	–	–	The LLDPE/OPP composite film showed antimicrobial activity against *Botrytis cinerea*	McKay et al., 2021
Polypropylene (PP) films impregnated by orange peel (OPe) extract	–	–	–	–	Extract addition confirmed by SEM, with a highly even distribution	–	Incorporation of antimicrobial activity against *S. aureus*	Casas Cardoso et al., 2022
Films based on lemon peel waste pectin (LPP) with purple sugarcane (*Saccharum officinarum*) peel anthocyanin extracts (PSPAEs)	Increase in the WVP with higher PSPAE concentrations	–	Decrease in the light transmittance of the films with PSPAE addition	Increase in the TS and EB values with higher PSPAE concentrations	Denser and more compact cross‐sectional structures with PSAPE addition; decrease in weight loss with higher PSAPE concentrations	No changes in the fast biodegradation of the films after PSAPE addition	Increase in the antioxidant activity of the films with higher PSAPE concentrations; color change (from pink to green) in the monitoring of shrimp and beef freshness	Jiang et al., 2022
Films of pectin from lemon and mango peel with silica	Decrease in the WVP with silica addition (1.25% w/v)	–	–	Increase in the TS and Young's modulus values and decrease in the toughness and EB with silica addition (1.25% w/v)	–	–	–	Karim et al., 2022
Films blended with persimmon (*Diospyros kaki* L.) and orange (*Citrus sinensis*) peel flour (OPF)	Increase in the water solubility with higher glycerol/OPF values	–	–	Increase in the TS values with higher OPF concentrations	The films were homogenous and thermally stable	–	Increase in the TPC and antioxidant activity of the films with higher OPF concentrations	Barone et al., 2023
Linear low‐density polyethylene (LLDPE) films modified with OPe (5%, 10%, 11.5%, and 12.5% w/w)	No changes in the WVP values with OP addition/increase in concentration	–	Increase in the yellowness and decrease in the light transmittance of the films with higher OP concentrations	Increase in the brokenness and brittleness of the films with higher OP concentrations	Decrease in the homogeneity of the microstructures with higher OP concentrations; no changes in thermal properties up to 11.5% of OP	–	–	Fehlberg et al., 2023
Active films of grapefruit pectin (GFPec) matrix plasticized with PEG400 with grapefruit peel methanolic extract (GFPE) and maltodextrin‐encapsulated lemon peel extract (MD‐LPE)	Increase (but statistically insignificant) in the water solubility and decrease in the WVP of the films with both GFPE and MD‐LPE	–	Decrease in the light transmittance of the films with GFPE addition	Increase in the TS values for the films containing both GFPE and MD‐LPE	Increase in the roughness and decrease in the homogeneity of the films with both GFPE and MD‐LPE addition; increase in the maximum temperature of degradation for the films with both GFPE and MD‐LPE	–	Films containing GFPE and MD‐LPE were effective in inhibiting the growth of *Escherichia coli* in tomatoes	Khalil et al., 2022
Cellulose‐based wrapping materials modified with AgNPs (0.5, 1, 2, and 3 mM) synthesized from pomegranate and kinnow peel extracts	Decrease in the WVP, swelling, and water content of the wrappers containing the AgNPs	Decrease in the oxygen permeability with the addition of AgNPs to the wrappers	Change in the color of the films from white to green and yellow with the addition of AgNPs from pomegranate and citrus peels	–	No significant aggregation of AgNPs in the cellulose microstructure	–	Increase in the antimicrobial activity of the cellulose wrappers containing the AgNPs (delayed occurrence of microbial count in bread packaged)	Gopalakrishnan et al., 2023
Bioelastomers of mandarin peel extract (MPE) and PDMS	Increase in the WVP of the bioelastomer with MPE addition/increase in concentration	–	Increase in the opacity and yellowness of the bioelastomer with MPE addition	Increase in the EB values with higher MPE concentrations	Change of a smooth to a porous surface of the bioelastomer with MPE addition	–	Increase in the antioxidant activity and antibacterial activity against gram‐positive (*S. aureus*), gram‐negative (*E. coli*), and antibiotic‐resistant bacteria (Methicillin‐resistant *S. aureus* and Vancomycin‐resistant *Enterococcus*) with higher MPE concentrations	Lee et al., 2023
Films based on sodium alginate (SA), pectin (PC), cellulose nanocrystals (CNCs), and anthocyanins extracted from red cabbage (RCA)	Decrease in the water solubility with CNC addition	–	Decrease in the transmittance of the films with CNC addition	Increase in the TS and EB values of the films with CNC addition	Increase in the thermal stability and the roughness of the films with CNC addition	–	Increase in the antioxidant activity with RCA increase; incorporation of color change in response to pH with RCA addition, not affected by CNC addition; change in color during the monitoring of shrimp freshness was affected by both the temperature and the position of the film (above or beside the samples)	Lei et al., 2023
Films of plasticized chitosan (pCS) with hydrolyzed orange peel (hOP) waste	Decrease in the WVP and water solubility values with hOP addition/increase in concentration	Decrease in the oxygen permeability with hOP addition/increase in concentration	Decrease in the transparency of the films with higher hOP concentrations	Increase in the elastic modulus and decrease in the EB values with higher hOP concentrations	Increase in the maximum temperature of degradation of the films with hOP addition	Acceleration in the biodegradation (62.9% weight loss after 26 days) in soil with higher hOP concentrations	Increase in the antioxidant activity, decrease in the antimicrobial activity against *S. aureus* and *E. coli*	Merino et al., 2023
Active multilayer films of functionalized pectin coated by polyhydroxyalkanoates (PHA) with spent coffee grounds (SCG)	Decrease in the WVP with the addition of SCG and PHA lamination	–	Increase in the opacity and decrease in the transparency of the films with SCG addition	Increase in the mechanical and strength and decrease in the flexibility of the films with PHA coating	Smoothness and homogeneity of the films with SCG confirmed by SEM; PHA layer presence detectable in the films with and without SCG	–	Increase in the antioxidant activity with higher SCG concentrations; films with SCG provided protection from carotenoid photodegradation in carrots; the multilayer film with SCG showed a wider inhibition halo against *Micrococcus luteus*	Mirpoor et al., 2023
Films based on OPe, orange pomace (OPo), and a combination of peel, pomace, and finisher pulp (OPPP)	OPo and OPPP films showed lower WVP values; no significant differences for the WCA values of the films	–	All films were yellowish and translucent; OPe film was the darkest one	OPo and OPPP presented higher TS and EB values	Rough and heterogeneous surfaces confirmed by SEM for all films	–	OPe film presented the highest TPC content; no significant changes in the antioxidant activity of the films	Santos et al., 2023
Chitosan‐based antioxidant biofilm with (5%, 10%, and 20% v/v) *Citrus limetta* pomace extract (LPE) and impregnated with halloysite nanotubes	Increase in the moisture content and solubility and decrease in the WVP with higher LPE concentrations	–	Significant changes in total color variation with higher LPE concentrations	Decrease in the TS and increase in the EB with higher LPE concentrations	Decrease in the homogeneity for the films containing the highest concentration of LPE	–	Increase in the antioxidant activity with higher LPE concentrations	Singhi et al., 2023
Films of pectin derived from citron peel with anthocyanins from *Phaseolus vulgaris* (dark red kidney bean) peels	Increase in the WVP with anthocyanins addition	–	Increase in the UV barrier properties with anthocyanin addition	–	Increase in the roughness and unevenness of the films with anthocyanins	–	Films with anthocyanins had antibacterial activity against *S. aureus* and *E. coli*; the films were used as a pH indicator and showed a significant color change from pink to brown on the quality test of chicken meat sample	Sudharsan et al., 2023
Pectin/PVA and pectin‐MgO/PVA films	Decrease in the WCA for the films with MgO nanoparticles	–	–	Increase in the TS values with MgO nanoparticle addition	Decrease in the weight loss for the films with MgO nanoparticles	Decrease in the degradation rate and percentages in both soil and water for the films with MgO nanoparticles	–	Suhasini et al., 2023
Films form the peel powder of four citrus fruits (orange, lemon, pomelo, and mandarin—OPP, LPP, PPP, and MPP)	Higher moisture contents for LPP and PPP films; lower WVP values for MPP and OPP films	Same tendency of WVP observed for oxygen permeability values	Lower transmittance in the visible light range for LPP and MPP films	No significant changes in the TS for the films; EB values higher for OPP and MPP films	Higher thermal stability for OPP and MPP films	–	Higher TPC and antioxidant activity for MPP and LPP films; all films showed reduced bacterial (*E. coli*, *S. aureus*, *Listeria monocytogenes*, *Salmonella typhimurium*) growth; all films showed slower increasing trends of PV and TBARS for corn oil in comparison with the unwrapped oil	Yun et al., 2023
Pectin/garlic (carbon dots, CDs) bionanohybrid films	No significant changes in the WVP and water solubility with CD incorporation	–	Decrease in the transmittance with CD incorporation	Increase in the TS and EB values with CD incorporation	Increase in the roughness and in the thermal stability of the films with CD addition	–	Increase in the DPPH antioxidant activity and in the antibacterial effect against *Salmonella* with higher CD concentrations; the films containing CDs retarded the loss of water in strawberries and prevented their contamination by foodborne microorganisms	Shen et al., 2024
Pullulan/bacterial cellulose (BC) film with (1%, 3%, 5%, 0% wt) *daidai* physiological fruit drop extracts (DE)	Decrease in the WCA of the films with higher DE concentrations; no significant changes in the WVP of the films	–	Increase in the yellowness and decrease in the transmittance of the films with DE addition/increase in concentration	Increase in the TS and decrease in the EB values with DE addition	All the film solutions were non‐Newtonian fluids with shear‐thinning properties; no significant changes in the decomposition temperatures of the films with DE addition	–	Increase in the antioxidant activity with higher DE concentrations; maintenance of the color and quality of strawberries coated by the film solutions	Xu, Ding, et al., 2024

Abbreviations: DPPH, 2,2‐diphenyl‐1‐picrylhydrazyl; EB, elongation at break; PV, peroxide value; SEM, scanning electron microscopy; TBARS, thiobarbituric acid reactive substances; TS, tensile strength; WCA, water contact angle; WVP, water vapor permeability.

### 6.1 Water‐related, gas barrier, light barrier, and optical properties

The performance of biopolymer films as food packaging materials is highly dependent on their water‐related properties (i.e., WVP, water solubility, and moisture uptake) and their barrier properties against gases and light (Sangroniz et al., 2019). Food packaging materials should have a minimum WVP to limit the interaction of water molecules with food and the environment. Moreover, solubility and swelling are also important factors since they influence the film's structural integrity in an aqueous environment. Santos et al. compared the properties of biomass‐based films obtained from the hydrothermal treatment of OPe, OPo, and OPPP (Santos et al., 2023). OPo and OPPP films showed improved water barrier properties. However, water contact angle (WCA) values indicated that all films were highly hydrophilic because of their composition rich in polysaccharides. Merino et al. found that the incorporation of hydrolyzed orange peel (hOP) waste improved the water resistance of the film (Merino et al., 2023). However, the observed values for WVP (1.5 × 10^−9^ g s^−1^ m^−1^ Pa^−1^) and solubility (∼50%) were higher than the observed for conventional polymers, which could limit the film applicability.

In order to reduce the high hydrophilic character of polysaccharide‐based films, different strategies have been pursued to enhance their barrier properties and water resistance. For instance, Mirpoor et al. reported the development of a multilayer film consisting of citrus pectin films coated with polyhydroxyalkanoates (PHA). This approach improved water sensitivity features, due to the intrinsic waterproofing characteristics of PHA, which significantly contributed to the increase in the WCA of the multilayer film (Mirpoor et al., 2023). The addition of nanomaterials, such as carbon dots (Shen et al., 2024), Ag (Gopalakrishnan et al., 2023), and SiO_2_ nanoparticles (Karim et al., 2022), has also been reported as an efficient way to improve the water‐related properties of packaging films, composed of citrus peel and garlic, cellulose, and pectin, respectively.

Packaging materials should also present strong barriers against oxygen, since excess gas can cause food oxidation and microbial deterioration. McKay et al. (2021) observed that the addition of orange peel powder (OPP) into linear low‐density polyethylene (LLDPE) could reduce the film oxygen permeability coefficient. The authors attributed this behavior to the presence of OPP agglomerates, which acted as a physical barrier to the oxygen molecules.

Optical properties are also important for the design of food packaging films. As consumers prefer to visualize the foods within their packaging, there is a high demand for transparent food films. However, direct exposure to light, especially UV light, degrades food quality through photolysis and photooxidation reactions (Merino et al., 2023). Therefore, it is necessary to develop packaging films that display suitable UV protective properties, appearance, and transparency. In general, the addition of citrus waste residues provides a color change in the films and reduces their transparency (Claudia Leites et al., 2021; Merino et al., 2023; Mirpoor et al., 2023). For instance, Claudia Leites et al. (2021) reported a decrease in the transmittance values of starch‐based films after the addition of orange residue, due to the presence of powder particles that were not solubilized. On the other hand, different authors stated that the incorporation of citrus waste components tends to improve the film light barrier performance due to the presence of compounds rich in C═O and C═C with strong light absorption in the UV region (200–400 nm) (Khalil et al., 2022; McKay et al., 2021; Santos et al., 2023).

### 6.2 Tensile, morphological, thermal and rheological properties

The mechanical properties of packaging are critical to ensure resilience to external stress, as well as to maintain barrier properties during its application. Key parameters such as TS (related to the resistance against tension forces), elongation at break (EB; stretching or deformation capacity), and Young's modulus (YM; rigidity of films) are usually measured to evaluate the mechanical performance of packaging films. Changes in tensile properties will mainly depend on the interaction between the components of the film and, consequently, the obtained structure (Barone et al., 2023). In this way, studies reporting enhancement or deterioration of mechanical properties by the incorporation of citrus waste have been reported.

For instance, Yun et al. (2023) demonstrated that the TS and EB of films made from orange, lemon, pomelo, and mandarin peel powders decreased as the content of soluble biopolymers diminished. The films with higher values of TS and EB also presented more compact inner structures, possibly due to the stronger interaction between the biopolymers. The influence of the resulting structure was also evaluated by Claudia Leites et al. (2021): by using orange residues in the powder and aqueous extract forms, different structures were obtained. When using the powder, a stiffer matrix was produced, which increased TS and YM values, whereas the aqueous extract led to a decrease in such parameters.

In general, mainly due to the rheological properties of pectin, it is observed that the incorporation of extracts obtained from citrus waste leads to a decrease in the TS and YM values and to an increase in the EB value. Singhi et al. showed, for example, that increasing the amount of *Citrus limetta* pomace extract from 0% to 20% gradually reduced the TS (from 14.47 to 4.76 MPa) and the YM (from 1658 to 1061 MPa) and increased the EB by 7% (Singhi et al., 2023). Also, an increase in the elastic modulus and a decrease in the EB by the progressive addition of hydrolyzed OPe to chitosan films were reported by Merino et al. (2023). To overcome the loss of mechanical quality of films, the use of other components that give rise to the desired properties is a frequently used strategy. In this regard, Suhasini et al. (2023) used inorganic MgO nanoparticles to obtain higher TS in their pectin/polyvinyl alcohol films, while Shen et al. (2024) used carbon dots to improve the TS (6.96 MPa) and EB (36.9%) of their original pectin films (3.62 MPa and 14.9%, respectively).

In addition to the mechanical aspects, the morphology of the film is also important. Since it is related to the roughness and thickness of the film, morphology is vital for predicting the interaction of the packaging with the food and ensuring adequate barrier properties. Furthermore, uniform distribution of active compounds in the polymeric matrix is essential to prevent agglomeration and guarantee their functionality throughout the entire film. Fehlberg et al. showed that increasing the OPe loading resulted in a higher number and larger agglomerates, which led to a decrease in the distance from each other, improving OPe distribution in the film (Fehlberg et al., 2023).

The viscosity of the film‐forming solutions is key in assessing the applicability of the produced films, as high viscosities can hinder their processability. Claudia Leites et al. (2021) showed that the addition of orange residues reduced the viscosity of the gel due to the substantial presence of acids, which favored starch gelatinization by reducing the size of the starch polysaccharide chains. Viscosity can also be a predictor of the film's mechanical properties. Xu, Ding, et al. (2024) showed that by increasing the amount of *Daidai* fruit drop extracts, the viscosity of the film also increased due to the interaction of the hydrophilic groups of pullulan and the phenolic acids or flavonoids, which resulted in better mechanical properties.

Thermal properties, typically assessed through thermogravimetric analysis (TGA), allow not only evaluating thermal stability of films but also obtaining information about the composition and the interaction between film components. Merino et al. (2023) demonstrated that the chemical interaction between plasticized chitosan (pCS) and hOP waste increased the thermal stability of the films and reduced their interaction with water. On the other hand, Han and Song (2020) showed that a decrease in thermal stability was observed after sage leaf extract addition, which was attributed to a less compact structure obtained.

### 6.3 Biodegradability, active and intelligent properties

One of the greatest challenges when developing food packaging materials is to replace the use of disposable fossil‐based plastics with renewable and biodegradable ones, which can be decomposed by living organisms (like microbes). The biodegradation process leads to products such as water, carbon dioxide, and biomass, and it can be affected by the inclusion of citrus wastes to biopolymers (Suhasini et al., 2023).

Merino et al. (2023) developed films of plasticized chitosan containing hOP and performed in‐soil biodegradability studies for 26 days. The authors found weight losses between 57% and 78%, increasing according to the increase in hOP concentration; the greater biodegradability of biopolymer‐based films (when compared to polyesters such as PLA) was attributed to the greater moisture content and amount of nutrients in the former, which allow microorganisms to colonize the film surfaces, accelerating biodegradation.

As pointed out in Section 2, the main properties highlighted in this review for active packaging are the antioxidant and antimicrobial ones, which will reduce the oxidation of food components (like lipids and pigments) and its contamination by microorganisms, respectively. The presence of phenolic compounds in active packaging is a strategy to confer antioxidant activity to polymers that, in their neat form, may not possess it. Many methods can be employed in order to quantify the scavenging activity of the developed materials, according to the radical or species to be scavenged/reduced. Han and Song (2020), for instance, prepared films of mandarin peel incorporated with phenolic extracts of sage leaf; the authors evaluated the antioxidant activity of the films by the ABTS (3‐ethylbenzothiazoline‐6‐sulfonic acid), DPPH, ferric‐reducing antioxidant powder (FRAP), and ferrous ion chelating (FIC) methods. All four methods showed that the leaf extract imparted concentration‐dependent antioxidant activity to the pectin films.

Yun et al. (2023) analyzed the antioxidant activity of their films regarding their ability to retard the oxidation and extend the shelf life of edible oils. The films acted as pouches, wrapping the oils, and the peroxide values (PVs) and thiobarbituric acid reactive substances (TBARS) were analyzed for 10 days. PV and TBARS are indicators of oils oxidation, since they represent the main products from primary and secondary oxidation stages, respectively. All their films were able to slow down the oxidation of the wrapped oil, when compared to an unwrapped one (control).

**FIGURE 4:**
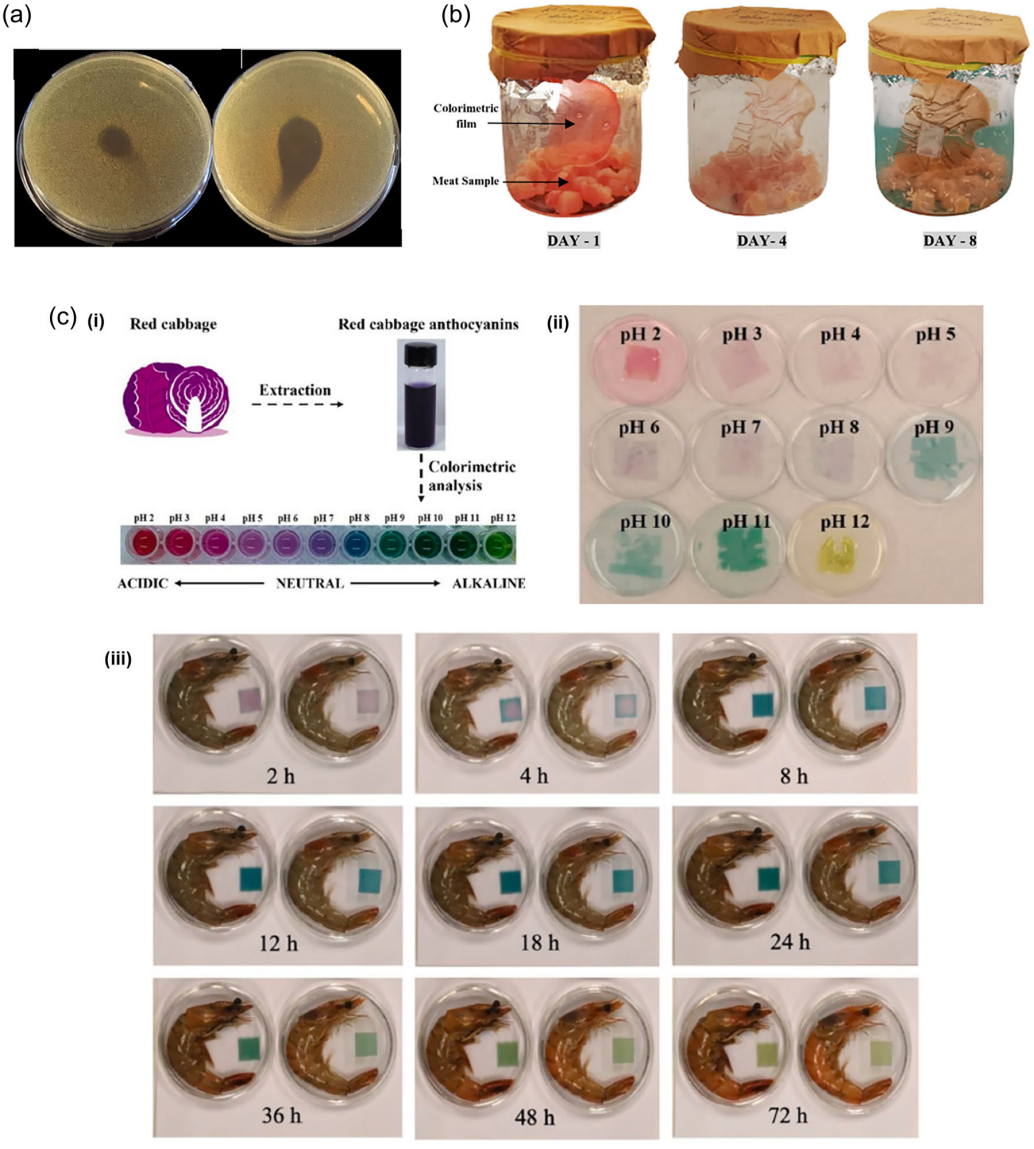
(a) Halo of inhibition assay of neat and multilayer active films of citrus pectin/PHA containing spent coffee ground against *Micrococcus luteus* (Mirpoor et al., 2023) (reprinted with permission of Elsevier, 2024) and (b) films of citrus pectin with anthocyanins from *Phaseolus vulgaris* peels to monitor chicken meat freshness (Sudharsan et al., 2023) (reprinted with permission of Enviro Research Publishers, 2024); (c) (i) color change in anthocyanins extracted from red cabbage in different pH, (ii) films of sodium alginate, citrus pectin, and cellulose nanocrystals containing these anthocyanins, and (iii) the application of these films in the shrimp freshness monitoring (Lei et al., 2023) (reprinted with permission of Elsevier, 2024).

The halo of inhibition assay is one of the most applied and simplest methods to evaluate the antimicrobial/antibacterial activity of active food packaging. For example, Mirpoor et al. (2023) developed active multilayer films of pectin coated by PHA with spent coffee grounds (SCG); the authors could not detect inhibition halo for the neat films (i.e., the pectin and PHA ones), but reported that the multilayer film containing SCG showed a larger inhibition halo against *Micrococcus luteus* after 10 days, which attested that SCG imparted antimicrobial activity to the films (Figure 4a).

The antibacterial effect of active packaging can also be evaluated in food models: Khalil et al. (2022) developed active films of grapefruit pectin with grapefruit peel methanolic extract and maltodextrin‐encapsulated lemon peel extract. The authors reported that the growth of *E. coli* in tomatoes wrapped with the developed materials containing both extracts was inhibited. Gopalakrishnan et al. (2023), in turn, prepared cellulose wrappers using green synthesized silver nanoparticles (AgNPs) with pomegranate and citrus peel extracts and reported a delayed occurrence of microbial count in packaged bread.

Following the discussion of Section 2, one of the most used types of intelligent packaging is the pH sensors, which can visually respond to pH variations during food spoilage. One of the most applied strategies to confer intelligent property to biopolymeric films is by incorporating molecules sensitive to pH variations, like anthocyanins. Examples of intelligent packaging based on citrus sources can be found in the literature: Sudharsan et al. (2023) prepared films of pectin derived from citron peel with anthocyanins from *Phaseolus vulgaris* peels. After evaluating the color change of anthocyanins in three different media (acidic, neutral, and basic), the authors applied the films as pH indicators in quality tests of chicken meat samples; the materials showed a color change from pink to brown, which the authors attributed to the presence of ammonia and amines (volatile nitrogen compounds) resulting from the breakdown of proteins and other macromolecules in meat during its deterioration (Figure 4b).

Lei et al. (2023) developed films of sodium alginate, pectin, CNCs, and anthocyanins extracted from red cabbage; after evaluating the color changes in the anthocyanins (Figure 4c) and in the films containing them (Figure 4d), the authors applied the materials as pH sensors for monitoring the freshness of shrimps. They reported that the color variation was affected by both the temperature and the position of the film (above or beside the samples) (Figure 4e). When positioned above the shrimp, the indicators exhibited great potential for monitoring the released volatile compounds.

## 7 FINAL CONSIDERATIONS AND FUTURE TRENDS

Citrus peels, pulps and seeds are major by‐products of citrus processing, yet they remain underutilized despite their rich composition. The development of food packaging materials based on or containing citrus waste has been growing in the last decade. In this review, we highlighted the latest advances in the field by focusing on the role, origins, types, and extraction methods of key components of citrus waste to be used in the development of food packaging materials. We also examined how these compounds specifically influence the critical physical–chemical and functional (active/intelligent) properties of the packaging materials. Some tendencies stand out among the studies covered by this review:

‐ Pectin is the most used citrus polymer in the production of food packaging films, and its properties have been often improved with the addition of other polymers or reinforcement agents; the other citrus polymers, such as cellulose and lignin, remain relatively underexplored.

‐ Casting is still the most used technique in the production of films, although electrospinning stands out as an important tool for producing materials with encapsulated citrus EOs.

‐ The vast majority of developed materials are intended to be applied as active films, and fewer studies have explored the preparation of citrus waste‐based intelligent materials.

Despite the extensive physical–chemical characterization of the developed materials, few studies actually applied them to food matrices in order to evaluate their active/intelligent properties.

Moreover, some challenges can be pointed out regarding the use of citrus waste as alternative sources of active and intelligent materials for food packaging purposes; these challenges lead to novel investigation perspectives and new insights into the development of packaging materials based on the reuse of citrus waste, as follows:

‐ The usage of citrus waste as raw materials in the development of packaging requires strict standardization to minimize variations in the composition and properties of the final material, ensuring minimum loss and degradation, as well as the safety of the material for packaging applications. Several regulatory bodies such as the US Food and Drug Administration (FDA) have been establishing regulatory standards for the use of packaging materials (Yao et al., 2024).

‐ In addition to standardizing the material, the final packaging cost is also extremely important for its acceptance by consumers. Generally, the cost of the packaging material should not exceed 10% of the total cost of the food (Oun et al., 2023). Adding value to citrus waste and using novel techniques to obtain packaging components can be a potential route to ensure that the final cost of the material is feasible.

Some important companies started to commercialize packaging materials made from plant‐based polymers, such as the Tetra Rex from Tetra Pak and the TopScreen from Solenis. These materials are appealing for being biodegradable, recyclable, and made from natural polymers, therefore highlighting citrus waste as a potential and innovative source for this type of application.

‐ One of the biggest challenges nowadays is the accumulation of microplastics in the environment, as well as their potential contamination in food due to the indiscriminate use and improper disposal of plastic food containers. Conventional plastic packaging that has been used for decades can promote the release of these microplastics in the food, often unnoticed by consumers (Jadhav et al., 2021). Food packaging materials based on natural polymers originated from citrus waste might be a suitable alternative to circumvent this problem.

Finally, based on the recently reported articles and the limitations identified, one can infer that it is still necessary to improve the performance of films regarding their use as packaging materials. In particular, their mechanical properties must be enhanced to achieve a performance level comparable to that of plastic films commercially available nowadays. In addition, there will be an increasing tendency to demonstrate in scientific studies real applications of these packaging for food storage.

Another area that should receive increased attention is the development of intelligent packaging systems. To advance in this direction, new materials and technologies still underexplored will need to be investigated. In this regard, the integration of nanotechnology with tools such as artificial intelligence (AI) and the Internet of Things (IoT) could greatly enhance the monitoring capabilities of both the food product and the packaging throughout the entire supply chain (Abekoon et al., 2024; Li et al., 2023). Mastering these new technologies and the use of advanced materials will also enable the customization of packaging to suit various restricted food storage conditions, such as different levels of humidity, temperature, and light exposure. Thus, large‐scale extraction and processing of citrus waste could support the commercial production of active and intelligent packaging based on citrus waste, facilitating the effective establishment of this industry in the future.

## AUTHOR CONTRIBUTIONS

**Mirella R. V. Bertolo**: Conceptualization; writing—original draft; writing—review and editing. **Tamires S. Pereira**: Writing—original draft. **Francisco V. dos Santos**: Writing—original draft. **Murilo H. M. Facure**: Writing—original draft. **FabrÃ­cio dos Santos**: Writing—original draft. **Kelcilene B. R. Teodoro**: Writing—original draft. **Luiza A. Mercante**: Writing—original draft. **Daniel S. Correa**: Conceptualization; writing—review and editing; supervision.

## CONFLICT OF INTEREST STATEMENT

The authors declare no conflicts of interest.

## Supporting information




**Figure S1**. Representation of the general chemical structures of (A) pectin, (B) cellulose, (C) some sugars that compose hemicellulose, and (D) lignin.
**Table S1**. Main phenolic compounds found in citrus peels and their structural formulas.
