# Supplementary material for: Citrus wastes as sustainable materials for active and intelligent food packaging: Current advances
Source: Compr Rev Food Sci Food Saf. 2025 Mar 4;24(2):e70144. doi: 10.1111/1541-4337.70144 (PMC11929373; doi:10.1111/1541-4337.70144)
Supplement: Supplementary file 1 — Figure S1. Representation of the general chemical structures of (A) pectin, (B) cellulose, (C) some sugars that compose hemicellulose, and (D) lignin. Table S1. Main phenolic compounds found in citrus peels and their structural formulas. [file CRF3-24-e70144-s001.pdf]

Supplementary Material

Citrus wastes as sustainable materials for active and intelligent food packaging: current advances

Mirella R. V. Bertolo[a*], Tamires S. Pereira[a,b], Francisco V. dos Santos[a,c], Murilo H. M. Facure[a], Fabrício dos Santos[a], Kelcilene B. R. Teodoro[a], Luiza A. Mercante[d], Daniel S. Correa[a,b,c*]

[a] Nanotechnology National Laboratory for Agriculture (LNNA), Embrapa Instrumentation, Sao Carlos 13560-970, SP, Brazil

[b] PPGQ, Department of Chemistry, Center for Exact Sciences and Technology, Federal University of Sao Carlos (UFSCar), 13565-905 Sao Carlos, SP, Brazil

[c] PGrCEM, Department of Materials Engineering, Sao Carlos School of Engineering, University of Sao Paulo, Sao Carlos 13563-120, SP, Brazil

[d] Institute of Chemistry, Federal University of Bahia (UFBA), Salvador 40170-280, BA, Brazil

*Corresponding authors: mirella.bertolo@alumni.usp.br, daniel.correa@embrapa.br

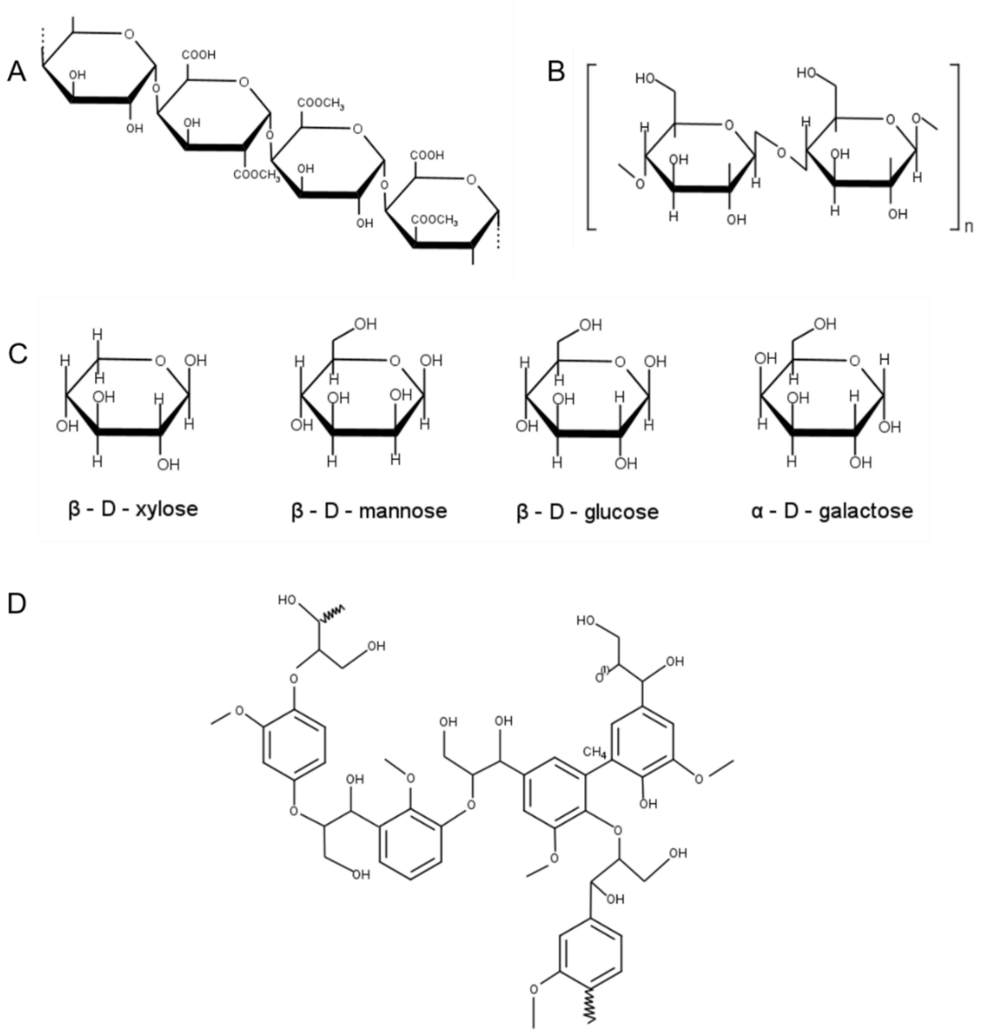

Figure S1. Representation of the general chemical structures of (A) pectin, (B) cellulose, (C) some sugars that compose hemicellulose, and (D) lignin.

Table S1. Main phenolic compounds found in citrus peels and their structural formulas.

Phenolic compound	Structural formula	Phenolic compound	Structural formula
Caffeic acid	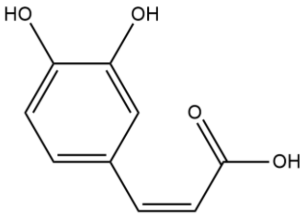	Didymin	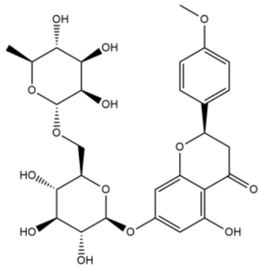
Chlorogenic acid	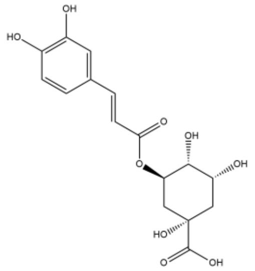	Diosmin	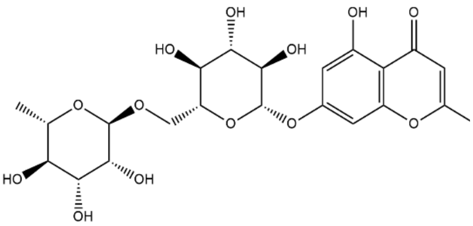
Feluric acid	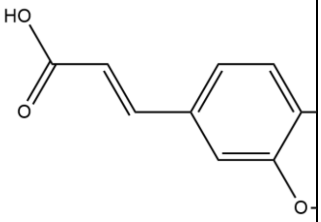	Hesperidin	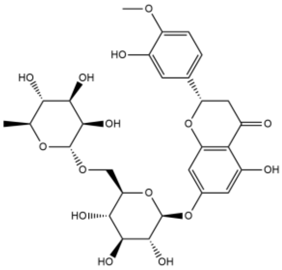
Gallic acid	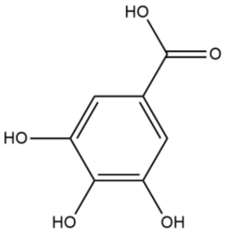	Hesperitin	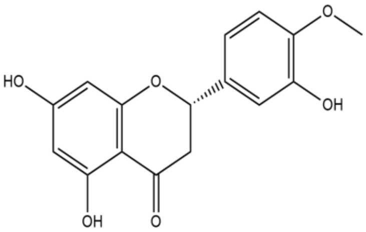
p-Coumaric acid	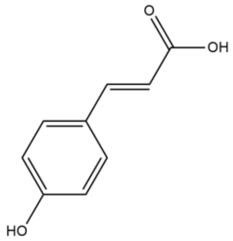	Kaempferol	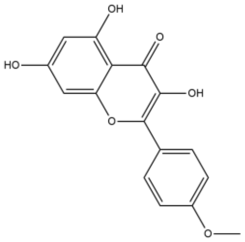
Sinapic acid	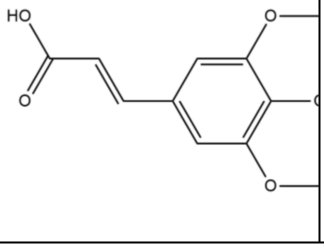	Naringenin	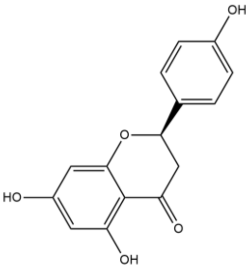

Vanillic acid	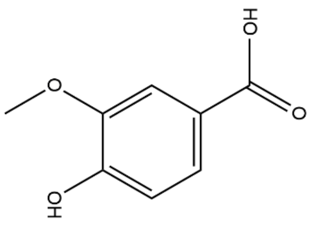	Narirutin	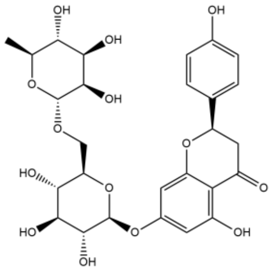
Naringin	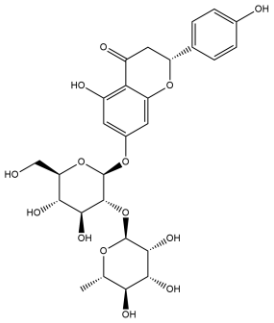	Quercetin	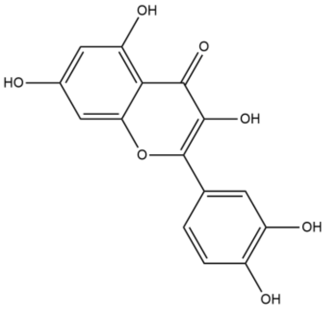
Rutin	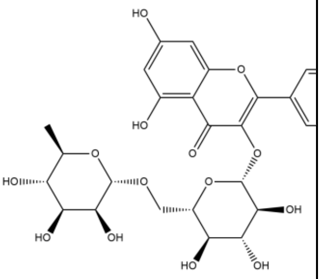	Neohesperidin	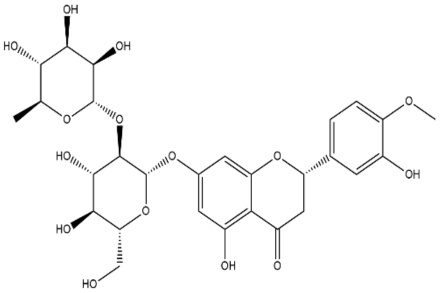
Sinensetin	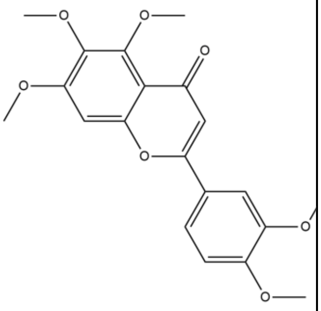	Neoeriocitrin	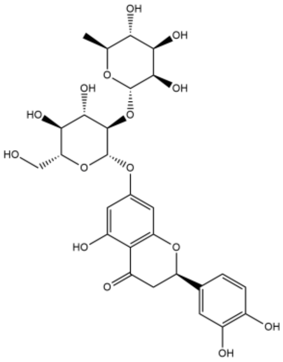
Tangeritin	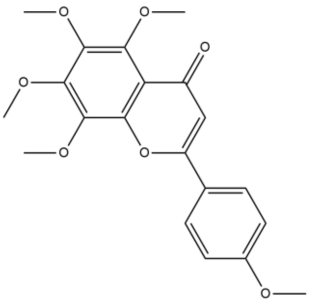	Poncirin	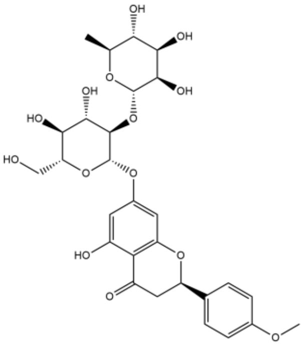
